# Plant-Derived Compounds as Promising Therapeutics for Vitiligo

**DOI:** 10.3389/fphar.2021.685116

**Published:** 2021-11-11

**Authors:** Yaobin Pang, Shi Wu, Yingjie He, Qing Nian, Jing Lei, Yejing Yao, Jing Guo, Jinhao Zeng

**Affiliations:** ^1^ Dermatological Department, Hospital of Chengdu University of Traditional Chinese Medicine, Chengdu, China; ^2^ Geriatric Department, Hospital of Chengdu University of Traditional Chinese Medicine, Chengdu, China; ^3^ TCM Regulating Metabolic Diseases Key Laboratory of Sichuan Province, Hospital of Chengdu University of Traditional Chinese Medicine, Chengdu, China

**Keywords:** vitiligo, oxidative stress, natural product, melanocytes, flavonoids, phenols, glycosides

## Abstract

Vitiligo is the most common depigmenting disorder characterized by white patches in the skin. The pathogenetic origin of vitiligo revolves around autoimmune destruction of melanocytes in which, for instance, oxidative stress is responsible for melanocyte molecular, organelle dysfunction and melanocyte specific antigen exposure as well as melanocyte cell death and thus serves as an important contributor for vitiligo progression. In recent years, natural products have shown a wide range of pharmacological bioactivities against many skin diseases, and this review focuses on the effects and mechanisms of natural compounds against vitiligo models. It is showed that some natural compounds such as flavonoids, phenols, glycosides and coumarins have a protective role in melanocytes and thereby arrest the depigmentation, and, additionally, Nrf2/HO-1, MAPK, JAK/STAT, cAMP/PKA, and Wnt/β-catenin signaling pathways were reported to be implicated in these protective effects. This review discusses the great potential of plant derived natural products as anti-vitiligo agents, as well as the future directions to explore.

## Introduction

### Pathogenesis of Vitiligo

Vitiligo is a chronic autoimmune destruction of melanocytes, leading to the pigment loss on the surface of skin and mucosa and then the gradual expansion of decolorized skin plaque (Seneschal et al., 2021). The severity of the disease affects about 1% of humans (Whitton et al., 2015). Clinically, vitiligo is divided into segmental vitiligo (SV), non-segmental vitiligo (NSV) and mixed vitiligo (MV) (Ezzedine et al., 2015). NSV is the most common type of vitiligo. Its clinical features are clear boundary, reticular, different size, different distribution, depigmentation and milky white. SV is a piece or several pieces, along the skin area dominated by a certain cutaneous ganglion segment, generally unilateral, accounting for 5–16% of all vitiligo cases (Speeckaert et al., 2020). MV includes the combination of SV and then bilateral NSV plaque after a period of time (Ezzedine et al., 2015). The debilitating nature of vitiligo leads to poor quality of life and mental health (Patel et al., 2017). Dermal exposure to UV light from depigmented skin increases the risk of skin irritation and cancer (Ahluwalia et al., 2017).

**FIGURE 1:**
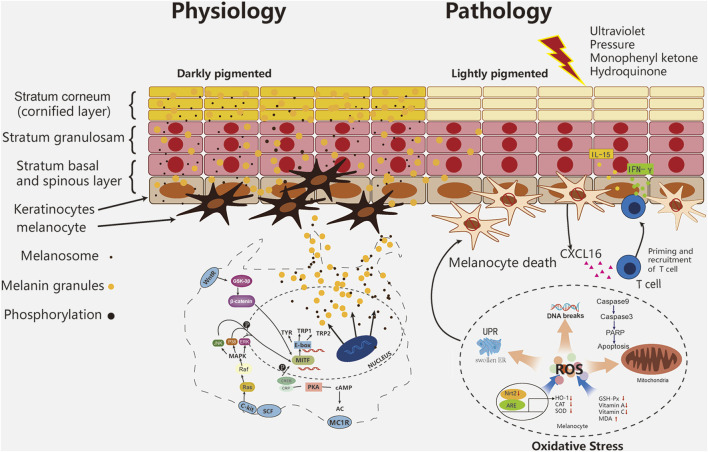
Melanocyte pathophysiology in vitiligo.

The main theories of the pathogenesis of vitiligo are: 1) oxidative stress theory, 2) autoimmune theory, 3) neural theory and 4) biochemical theory. The autoimmune theory is the most accepted one (Rodrigues et al., 2017). More than half of the 40 susceptibility genes revealed by genome-wide analysis are involved in immunoregulatory activities (Jin et al., 2016). Strong evidence shows that oxidative stress is a key factor in the occurrence and development of diseases (Denat et al., 2014). Several endogenous and exogenous stimuli are related to the occurrence of diseases. Endogenous factors include melanin synthesis, proliferation, differentiation, cell metabolism, immune response and apoptosis (Al-Shobaili and Rasheed 2015). Exogenous stimuli include exposure to the environment (e.g., cytotoxic chemicals, trauma, UV exposure, monophenones and other phenolics), other diseases (severe infections, neurological disorders, malignancies, calcium imbalance), and pharmaceutical applications (e.g., certain hormones, vaccination, drugs) (Xie et al., 2016). These all induce oxidative stress in melanocytes, which may be important in activating autoimmune responses associated with vitiligo (Abdel-Malek et al., 2020; Chen X. et al., 2019; Xie et al., 2016). The decrease of the level and activity of antioxidant enzymes (such as catalase and glutathione peroxidase) and the imbalance of Pro oxidation/anti oxidation balance are also the reasons for the production and accumulation of ROS (Wang Y. et al., 2019). Nrf2-ARE regulates cellular protective genes related to oxidative stress (Jian et al., 2011). In vitiligo melanocytes, Nrf2 has nuclear translocation and decreased transcriptional activity, resulting in decreased HO-1 expression and abnormal redox balance (Jian et al., 2014). Due to the deficiency of antioxidant function, melanocytes in vitiligo are particularly sensitive to ROS accumulation, which leads to DNA damage, protein oxidation/breakage, mitochondrial dysfunction, endoplasmic reticulum abnormalities and lipid peroxidation (Bickers and Athar 2006; Chen J. et al., 2021). Increased ROS levels even modified tyrosinase (Tyr) and other melanin proteins into new antigens (Rodrigues et al., 2017). Oxidative stress leads to the accumulation of misfolded proteins in the lumen of the endoplasmic reticulum (ER), which in turn activates the unfolded protein response (UPR) to restore cellular homeostasis and maintain cell survival. The disturbance of endoplasmic reticulum Ca2+ triggered by oxidative stress may also induce UPR and apoptosis (Carreras-Sureda et al., 2018). Under continuous cell pressure, UPR promotes autoimmune response through apoptosis cascade, and then activates CD8^+^ T cells to produce adaptive immune response, and T cells release interferon-γ (INF-γ), It binds to receptors on keratinocytes, further releases and presents inflammatory cytokines such as CXC-L16 and IL-15, and further recruits T cells to the skin through a positive feedback loop (Bergqvist and Ezzedine 2021). The recruitment of CD8^+^and T cells induced by cytokines and chemokines ensure the final destruction of epidermal melanocytes (Figure 1). After naive T cells are activated by antigen-presenting cells, a small subset of these precursor cells eventually develop into several subsets of memory T cells, including effector memory T (T_EM_) cells, tissue resident memory T (T_RM_) cells and central memory T (T_CM_) cells. T_RM_ play a major role in vitiligo recurrence (Chen and Shen 2020). In the process, melanocytes, fibroblasts, innate lymphoid cells, natural killer cells, and keratinocytes collectively contribute to the pathogenesis of vitiligo (Seneschal et al., 2021).

### Vitiligo Therapy

However, there is no clear treatment for local and systemic vitiligo. The most widely used therapy is local steroid and narrowband ultraviolet B monotherapy (Grimes and Nashawati 2017), they are not effective in all patients and are expensive, not easily accepted, and are associated with side effects (Huo et al., 2014). Local corticosteroids, calcineurin inhibitors and phototherapy are still the basis of treatment, and long-term use of steroids can lead to decreased immunity (Whitton et al., 2016). Among the numerous treatments currently available, including medical, physical, or surgical approaches, each modality has its disadvantages and side effects (Yamaguchi et al., 2007).

**FIGURE 2:**
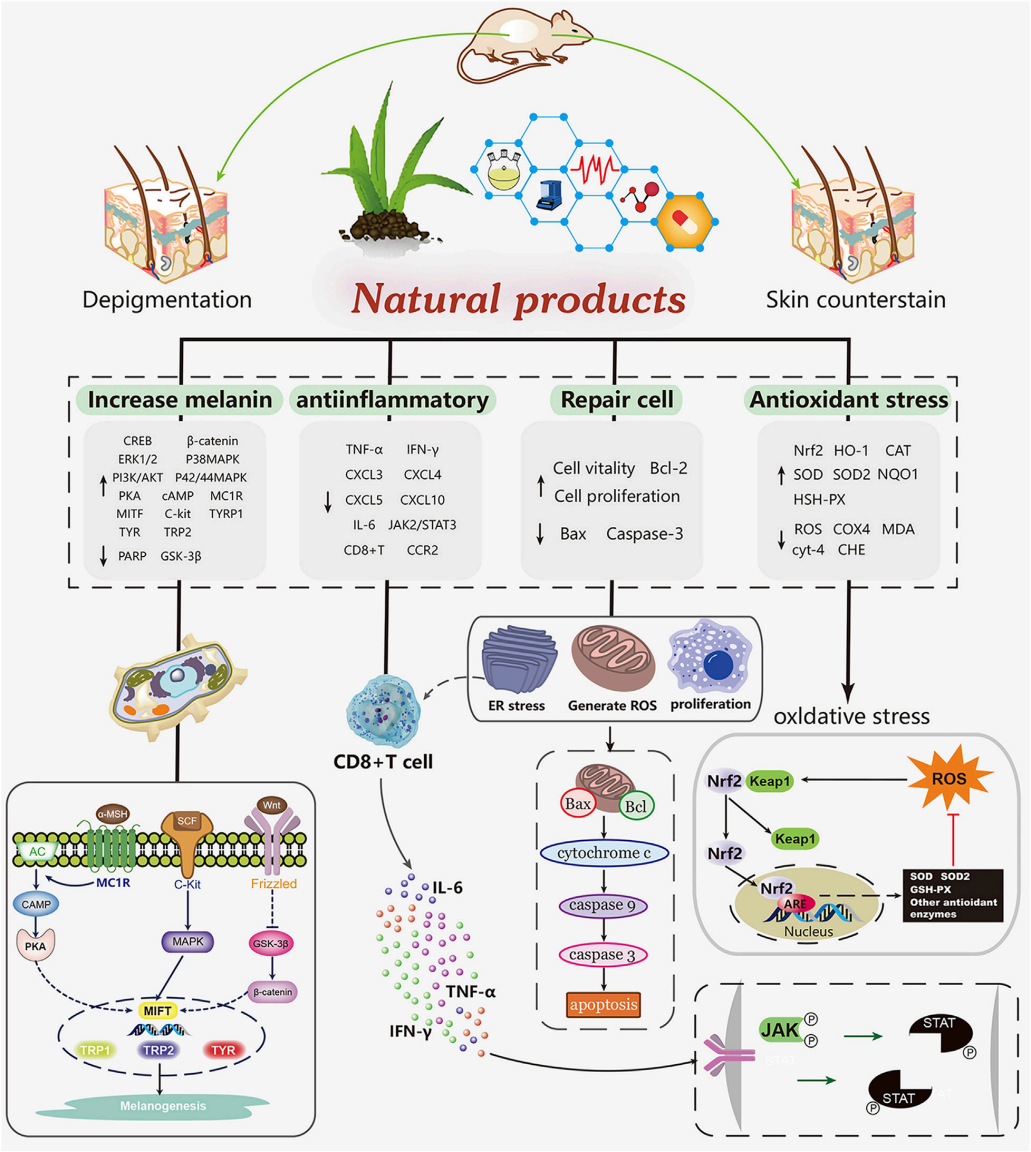
Mechanism of natural products in the treatment of vitiligo.

*In vivo* and *in vitro* experiments, natural products have been shown to promote melanin production and prevent melanin from being destroyed in a network way. It mainly includes scavenging free radicals (NOS) to alleviate the damage of melanocytes caused by oxidative stress, activating melanogenesis related pathways, increasing the expression of tyrosinase gene, reducing the expression of chemokines and inflammatory cytokines, preventing the migration of CD8 + T cells. This paper reviews several natural drugs for the treatment of vitiligo. Among the natural products that we screened, 16 compounds (such as baicalein, quercetin, paeonol. etc) exert antioxidant effects to protect melanocytes by scavenging free radicals, activating the Nrf2/HO-1 pathway, while maintaining normal cell morphology, slowing down apoptosis and preventing injury. Four compounds (vitexin, baicalin, EGCG and berberine) achieved repigmentation of vitiligo skin lesions via anti-inflammatory effects, the process of which involved the activation of the JAK/STAT pathway as well as the inhibition of autoimmunity caused by the migration of immune cells such as CD8 + T. In mammals, there are three major melanocyte specific enzymes catalyzing melanin biosynthesis: tyrosinase (TYR), tyrosinase associated protein 1 (TRP-1) and TRP-2. TRP-1 and Trp-2 are downstream functional proteins of TYR (Yin et al., 2018). Microphthalmia associated transcription factor (MITF) is a transcription factor important for melanogenesis genes (Hearing 1999). Previous studies summarized three main pathways of melanin biosynthesis regulated by tyrosinase: MAPK, cAMP/PKA, Wnt/β - catenin signaling pathway, and reviewed the effects of several natural products on melanin synthesis and tyrosinase activity (Niu and Aisa 2017; Pillaiyar et al., 2017). Among the compounds we screened, 37 compounds (such as quercetin, afzelin, puerarin, geniposide, etc) promote melanocyte generation through the above pathways (Figure 2).

### Flavonoids

#### Baicalein

**FIGURE 3:**
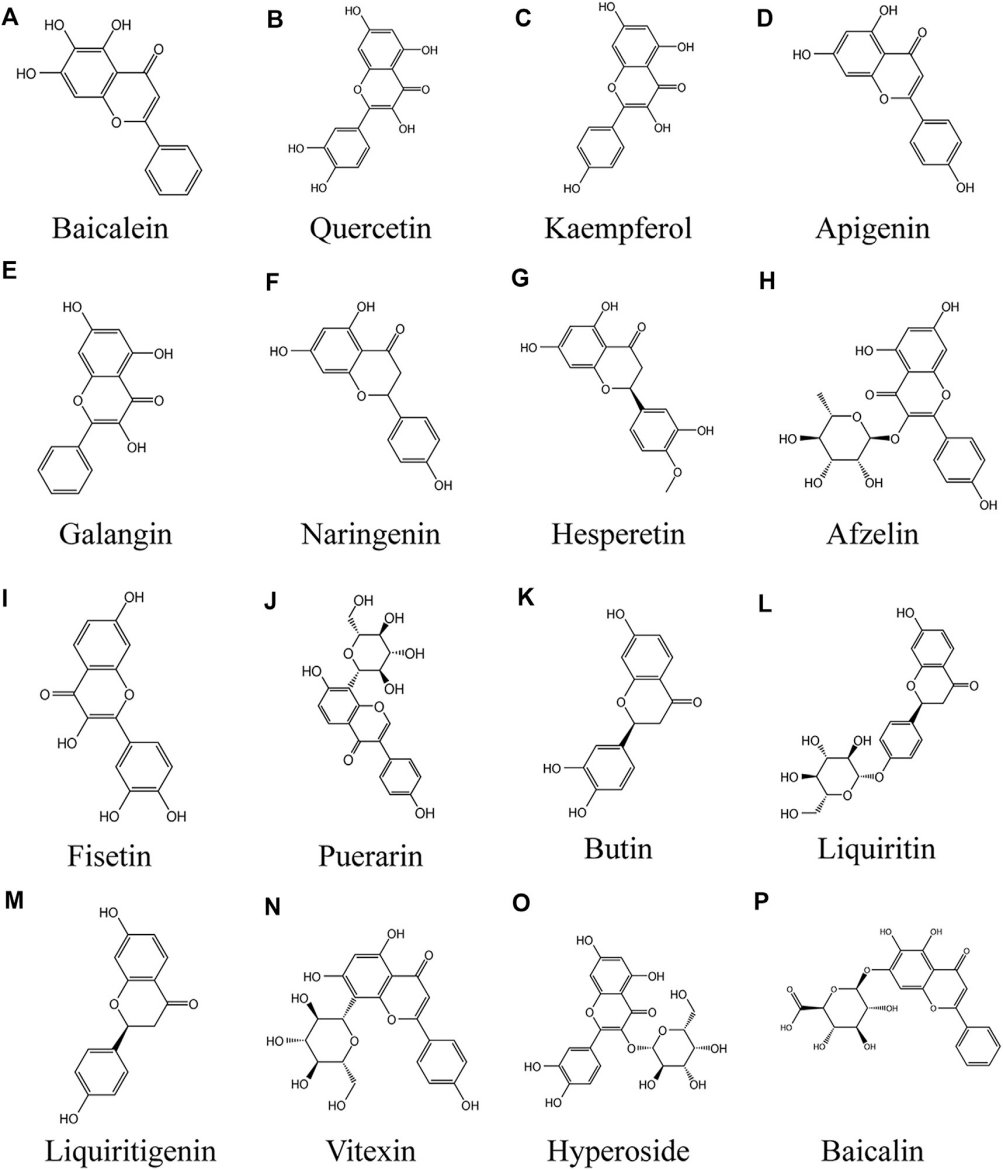
Anti vitiligo flavonoids **(A)** Baicalein, **(B)** Quercetin, **(C)** Kaempferol, **(D)** Apigenin, **(E)** Galangin, **(F)** Naringenin, **(G)** Hesperetin, **(H)** Afzelin, **(I)** Fisetin, **(J)** Puerarin, **(K)** Butin, **(L)** Liquiritin, **(M)** Liquiritigenin, **(N)** Vitexin, **(O)** Hyperoside, **(P)** Baicalin.

Baicalein is a flavonoid extracted from the roots of Scutellaria baicalensis Georgi that has been extensively applied in Traditional Medicine in Asia (Figure 3A) (Kim et al., 2014). It has been reported to have anti-cytotoxic, anti-inflammatory, and anti-tumor effects (Huang et al., 2005; Hwang et al., 2005; Yarla et al., 2016). In addition, the antioxidant effects of baicalein have received special attention during the past decades, including reducing the levels of reactive oxygen species (ROS) generated by chemical agents (Chiu et al., 2010; Choi et al., 2016; Zhao et al., 2018) or ultraviolet radiation (Wang et al., 2018). Baicalein has strong antioxidant properties, partly because compounds scavenge ROS by oxidative consumption of the three 5, 6, 7-position OH–groups in its structure (de Oliveira et al., 2015), and it form stable semiquinone radicals, which also underlie its powerful antioxidant activity (Gao et al., 1999). The apoptosis of PIG1 cells induced by H_2_O_2_ may be mediated by mitochondrial pathway. In the *in vitro* model of H_2_O_2_-induced oxidative stress of PIG1, baicalein protected PIG1 cells from H_2_O_2_-induced oxidative stress and apoptosis, maintained mitochondrial membrane potential, released cytochrome c, and decreased the Bax/Bcl-2 ratio. The mechanism mainly involves the activation of mitochondrial dependent caspase and the regulation of p38MAPK pathway. Baicalein at the concentration of 40 µM had the strongest protective effect on melanocytes (Liu et al., 2012). In human vitiligo melanocytes (PIG3V) induced by hydrogen peroxide, baicalein increased the expression of Nrf2 and its downstream gene HO-1 in PIG3V cells, promoted the translocation of Nrf2 from cytoplasm to nucleus, indicating that the protective effect of baicalein on melanocytes depends on Nrf2 signaling pathway (Ma et al., 2018). Baicalein also has antioxidant effect on keratinocytes (Wang et al., 2018), therefore, the development of baicalein topical preparation for the treatment of vitiligo may be a feasible method.

### Quercetin

Quercetin is a kind of polyhydroxy flavonoid, which is chemically named 3,3,4,5,7-pentahydroxyflavone (Figure 3B). It has high content in apple, onion and green tea, and also exists in *Asparagus racemosus* Willd., Ficus ingens (Miq.) Miq., Coriandrum sativum L. and Capparis spinosa L. It is one of the most consumed flavonoids in people's daily diet (Alvarez-Arellano et al., 2020). Quercetin has a variety of biological activities, such as antioxidant and scavenging free radicals (Wang et al., 2021e), anti-cancer, anti-aging (Zhu et al., 2015), anti-inflammatory (Rauf et al., 2018; Cui et al., 2019) anti-virus and immune regulation (Zhu et al., 2015; Alvarez-Arellano et al., 2020). It has a very important clinical significance in the treatment of bacterial infection, viral infection, hyperlipidemia and immune system diseases, especially for those caused by increased oxidative stress Cell damage and even mitochondrial dysfunction related diseases have potential therapeutic effects. Quercetin treatment of cultured melanoma cells or NHEM promote melanin synthesis and tyrosinase activity. In cell experiment, treatment of HMVII cells with quercetin at different doses (1, 5, 10, 20 μm) and for different times (1, 3, 5, 7 days) resulted in a dose and time-dependent increase in melanin content. The mechanism of action may be reflected by the genomic mechanism of new messenger RNA and protein synthesis (Nagata et al., 2004). Interestingly, the triple combination of GT extract/quercetin/folic acid prevented H_2_O_2_-induced cell damage in a synergistic manner, suggesting that effective antioxidant combinations should be studied to combat ROS types. Among them, quercetin and GT extract had strong protective effect on H_2_O_2_ induced cell death, and 100 μm quercetin had the most significant protective effect (Jeong et al., 2005). Cuiping Guan et al. observed endoplasmic reticulum expansion and configuration changes in cells treated with H_2_O_2_ and NaOH/H_2_O_2_. Quercetin alleviated the increase of ROS level induced by H_2_O_2_, and weakened the inhibition of tyrosinase expression by hydrogen peroxide. The mechanism may be to prevent oxidative stress damage, and tyrosinase is effectively exported from endoplasmic reticulum (Guan et al., 2015). Different nanoparticles, such as transporter, solid lipid nanoparticles, nanostructured lipid carriers, liposomes, nano emulsions and polymer nanoparticles, maximize the ideal properties and/or therapeutic activity of quercetin (Nasr and Al-Karaki 2020), which provides a feasibility for the external treatment of vitiligo with quercetin.

### Kaempferol

Kaempferol (Figure 3C) is a kind of flavonoids, which mainly comes from the rhizome of Kaempferia galanga L. (Liu Z. Q. et al., 2021). It widely exists in all kinds of fruits, vegetables and beverages, hazelnut, tea, propolis, broccoli and grapefruit (Calderón-Montaño et al., 2011). Kaempferol can be used to combat cardiovascular disease, cancer outbreak, immune dysfunction, diabetes, oxidative stress and other diseases (Imran et al., 2019). Kaempferol has no obvious cytotoxic effect on B16F10 cells at the concentration of 16–32 μm for 24 h (Wang et al., 2017). Kaliziri is an extract from Baccharoides anthelmintica (L.) Moench, which has showed relatively good therapeutic effects for vitiligo (Maimaiti et al., 2017). Kaempferol is one of the main active components of *Baccharoides anthelmintica* (L.) Moench extract (Tuerxuntayi et al., 2014). After treatment with 1, 5, 10 and 20 μm kaempferol for 7 days, melanin content in hmvii cells increased significantly. It enhanced tyrosinase activity in HMVII cells and mouse buccal hair follicles by inducing tyrosinase protein expression (Takekoshi et al., 2014). Based on the SDTNBI method and experimental verification, kaempferol markedly increased tyrosinase activity and melanin biosynthesis gene expression in B16F10 cells, and effectively promoted melanin synthesis (Wang et al., 2017).

### Apigenin

Apigenin (4′, 5,7-trihydroxyflavone; (Figure 3D) natural plant flavone, widely exists in common fruits and vegetables. It is considered to be a flavonoid with biological activity (Liu et al., 2017). Compared with other flavonoids, apigenin is relatively non-toxic and non-mutagenic, and has significant effects on normal cells and cancer cells (Patel et al., 2007). It has demonstrated a variety of pharmacological effects, including antidepressant (Li et al., 2015; Zhang X. et al., 2019), anti-inflammatory, liver protection, antithrombotic, anticancer, anti-aging (Lim et al., 2015), anti-oxidant (Wang J. et al., 2020). Apigenin significantly increased the activities of glutathione peroxidase (GSH PX), catalase (CAT) and superoxide dismutase (SOD), in a dose-dependent manner, and significantly inhibited the level of malondialdehyde (MDA), a biomarker of oxidative stress, in a H_2_O_2_ induced cell line PIG3V. Interestingly, apigenin markedly increased protein expression levels of Nrf2 and its downstream NQO1 and HO-1 in a dose-dependent manner, but had no effect on Nrf2 knockout cells. The results showed that apigenin protects melanocytes from oxidative damage dependent Nrf2 pathway (Zhang et al., 2020). In the dopamine (DA)—induced melanocyte model, 10 μm apigenin treatment significantly reduced ROS aggregation, reduced Da induced melanocyte apoptosis, and inhibited caspase-3 and PARP activities, which may be involved in the anti-apoptotic effect of apigenin. The mechanisms include inhibition of JNK, p38MAPK and Akt (Lin M. et al., 2011). *In vitro*, HMVII cells were treated with apigenin at 1, 5, 10 and 20 μm for 7 days, and the melanin content increased significantly (Takekoshi et al., 2014). So far, there is little evidence that apigenin promote adverse metabolic reactions *in vivo* when ingesting nutrition related amounts (Shukla and Gupta 2010; Takekoshi et al., 2014; Weng et al., 2016). Apigenin's local drug delivery system transports apigenin to local skin tissue instead of penetrating into blood circulation (Li et al., 1996). Therefore, apigenin may be a relatively safe method for the treatment of vitiligo.

### Galangin

Galangin (GA, 3,5,7-trihydroxyflavone; Figure 3E) is an important natural active flavone, which is mainly extracted from the roots of Alpinia officinarum Hance, it has long been used as herbs and spices in South Africa and Asia (Yang et al., 2018). GA has been reported to possess a variety of biological activities, including antibacterial (Skiba et al., 2016), antiviral, anti-inflammatory (Cushnie et al., 2007), anti-obesity (Ma et al., 2019) and antioxidant (Sinha et al., 2014), it is reported that these effects are exerted by regulating NF - κ B, Nrf2 and cAMP/CREB signaling pathways (Yang C.-C. et al., 2020). 4.25 mg/kg GA significantly increased the number of basal melanocytes and melanoepidermal cells in shaving area of mice with hydroquinone induced vitiligo, and promote the melanin hair follicles to increase, the mechanism is to prevent oxidative stress by reducing cholinesterase (CHE) activity and MDA content and increase the expression of TYR protein. Malondialdehyde (MDA) is the final product of lipid peroxidation, which is considered as a specific indicator of oxidative stress (Huo et al., 2014). However, GA metabolism is fast and its bioavailability is low. 90% of GA is metabolized in 1 h and is metabolized in 2 h in hepatocytes. Therefore, it is necessary to modify GA by methylation to slow down its metabolism and improve its bioavailability (Fang et al., 2019).

### Naringenin and Hesperetin

Naringenin (Figure 2F) and hesperetin (Figure 3G) are two main flavonoids identified from Citrus × limon (L.) Osbeck extract (Singh et al., 2020). The flavonoids in sweet orange peel include flavonoid glycosides, flavones and flavonols, among which flavanones exist in the form of glycosides (hesperidin and naringenin) or aglycones (hesperidin and naringenin) (Ramful et al., 2010). Citrus flavonoids have many biological activities, such as anti-tumor, anti-oxidation and anti-inflammatory (Salehi et al., 2019). It was found that flavonoids from navel orange peel extract had antioxidant activity (Long et al., 2021). Citrus products stimulate cell melanin production and tyrosinase expression, thereby preventing skin damage caused by ultraviolet light (Chiang et al., 2011). Huang et al. (2012) used 20 μg/ml citrus extract for melanin synthesis experiment. Citrus are rich in hesperidin, neohesperidin or naringin, and acid hydrolytic extracts of these three species of Citrus promote melanin synthesis. In this experiment, 50 μm hesperetin increased the expression of β-catenin, induced the rapid phosphorylation of p38MAPK, ERK and Akt, and activated the downstream transcription factor CREB phosphorylation in less than 1 h. Hesperidin stimulates melanogenesis by activating CREB and MAPKs in Wnt/β-catenin pathway (Huang et al., 2012). Naringenin enhanced tyrosinase activity of B16 mouse melanocytes in a time-dependent manner and increased melanin content in a concentration dependent manner, reaching the maximum at 100 μM. The mechanism included increasing the expression level of melanin producing enzyme (TYRP1 and DCT) and MITF (Ohguchi et al., 2006). Another study also confirmed that naringenin up-regulated tyrosinase activity of B16-F10 cells in a concentration dependent manner. Naringenin up regulated the expression of MITF by increasing the expression of β-catenin and the phosphorylation of Akt or GSK3, and then increased the activity of tyrosinase, so as to improve the melanin synthesis of B16-F10 cells, rather than through cAMP pathway (Huang et al., 2011). These results suggest that hesperetin induced melanogenesis in cell models may contribute to the development of topical beauty agents.

### Afzelin

Afzelin (3-O-α-l-rhamnopyranoside; Figure 3H) is a flavonoid isolated from Thesium chinense Turcz. and widely distributed in Korea and China (Li G.-H. et al., 2021). Previous studies have shown that afzelin has antibacterial, anticancer and anti-inflammatory effects (Satthakarn et al., 2015). Afzelin markedly alleviated ultraviolet induced oxidative stress in human skin, damage of mitochondrial membrane potential and mitochondrial permeability (Shin et al., 2013). Afzelin showed strong anti-oxidant activity in DPPH radical scavenging experiment (Kim et al., 2008). Many studies have shown that afzelin could effectively treat skin diseases (Lee et al., 2014). In the experiment of melanogenesis induced by afzelin in human epidermal melanocytes, 100 μm afzelin increased the protein levels of TRP-1 and TYR by up regulating MITF, but did not increase the protein levels of TRP-2. The mechanism is p38MAPK phosphorylation, which up regulates MITF, and is independent of cAMP/PKA pathway (Jung et al., 2016).

### Fisetin

Fisetin (3,30,40,7-tetrahydroxyflavone; Figure 3I) is a dietary flavone, which exists in a variety of vegetables and fruits, including apples, strawberries, grapes, cucumbers and onions (Khan et al., 2018). Studies have found that fisetin has anti allergic, anti-arthritis and neuroprotective effects (Ahmad et al., 2017; Ahmad et al., 2019). New data also showed that fisetin had anti-cancer activity (Jie et al., 2015; Khan and Mukhtar 2015; Ding et al., 2020). In particular, it has recently been found that it inhibited inflammation and antioxidant stress (Silva et al., 2007; Ahmad et al., 2019). Interestingly, fisetin has a two-way regulatory effect on melanin production. Takekoshi et al. first reported that fisetin could promote Tyr activity and melanin content of human melanoma cells (Takekoshi et al., 2014). However, Shon et al. found that fisetin inhibited the melanin content in and out of mouse B16F10 melanoma cells mediated by α - MSH(Shon et al., 2016). A recent study found that the difference of dual effects of fisetin was related to its concentration, because high concentration of fisetin inhibited the synthesis of melanin in zebrafish larvae (400 μm) and B16F10 melanoma cells (40 μm). When the concentration of fisetin exceeded 25 μm, the inhibitory activity increased slightly, and 200 μ m had the highest inhibitory rate on mushroom tyrosinase activity *in vitro*. Surprisingly, 5 μm fisetin slightly increased the content of spontaneous melanin and extracellular melanin, fisetin (at 20 μm) markedly increased the content and release of melanin in B16F10 cells by up regulating the expression of TYR and MITF, and promoted the melanin synthesis of zebrafish larvae. The mechanism is that fistein inhibits GSK-3 β, which activates β - Catenin, which leads to melanogenesis by activating MITF and tyrosinase (Molagoda et al., 2020). Therefore, low dose of fisetin may be an effective drug in the treatment of vitiligo.

### Puerarin

Puerarin (7,40-dihydroxyisoflavone-8b-glucopyranoside; Figure 3J) is an isoflavonoid derivative isolated from the root of the traditional Chinese medicine *Pueraria lobata* (Willd.) Ohwi (Zhang 2019). Puerarin exhibits a wide range of antioxidant activities in cardiovascular diseases, diabetes, obesity, osteoporosis, and other diseases (Xu et al., 2021; Chang et al., 2021; Liu Y. et al., 2021; Xiao et al., 2020). In addition, puerarin has anti-inflammatory, anti-viral and other pharmacological activities (Wang Z.-K. et al., 2020; Wang H. X. et al., 2021). Puerarin exhibited obvious pharmacological activities against vitiligo *in vitro* and *in vivo*. Park et al. found that puerarin could increase the melanin content of melanocytes *in vitro*, and topical application could improve the melanin content of mouse skin tissue, the mechanism is via activation of the cAMP pathway, followed by elevation of MITF, tyrosinase, Trp-2, and Bcl-2 to increase melanocyte survival and melanin content (Park et al., 2014). In the 4-benzyloxyphenol-induced vitiligo mouse model, after one week of Puerarin Treatment, HE staining of the skin at the depigmented sites showed increased hair follicles, which were surrounded by a large number of melanocytes. Puerarin at 40 μmol/L significantly increased the melanin content of human melanocytes by decreasing the phosphorylation of ERK in the cells to promote TRP-1 and MITF expression, which led to an increase in melanin content (Ding et al., 2019).

### Butin

Butin (7, 30, 40-trihydroxydihydroflavone, BUT; (Figure 3K) flavonoid with antioxidant activity, isolated from *Alpinia officinarum* Hance*, Dalbergia odorifera* T.C.Chen (Duan et al., 2017). BUT exhibited a wide range of pharmacological activities for the treatment of aging, diabetes, liver diseases, and cancer (Zhang et al., 2011). In the hydroquinone-induced vitiligo mouse model, (4.25, 42.5 mg/kg) butin increased the melanin content in the skin lesions by increasing the expression of Tyr and TRP-1 protein, reducing the serum cholinesterase activity and malondialdehyde content. Besides, BUT promoted the proliferation of basal melanocytes (Huo et al., 2017). A recent study found that butin induced melanin production both *in vivo* and *in vitro* when the concentration was 40 μmol/L, while tyrosinase activity peaked. Meanwhile, in a H_2_O_2_-induced zebrafish model, butin reduced the levels of reactive oxygen species *in vivo* (Lai et al., 2021).

### Liquiritin and Liquiritigenin

Liquiritigenin (LQ; Figure 3L) and liquiritigenin (LQG; Figure 3M) are flavonoids extracted from *Glycyrrhiza uralensis* Fisch. ex DC. (Du et al., 2021; Mou et al., 2021). They have many biological activities, such as antiviral, anti-inflammatory, anti-oxidation, anti-tumor and so on (Pastorino et al., 2018). LQ and LQG were used to treat mouse melanoma B16-F1 cells and human melanoma hmvii cells with different doses for 72 h. The results showed that both natural drugs significantly increased the content of melanin in melanocytes in a dose-dependent manner, and had no effect on cell viability. Interestingly, both LQ and LQG could significantly up regulate tyrosinase activity and the expression of MITF and its downstream TRP-1 and Trp-2. Further studies have found that 50 μm LQ and LQG trigger melanin synthesis through p38 phosphorylation and activation of PKA/CREB signaling pathway (Uto et al., 2019).

### Vitexin

Vitexin (apigenin-8-C-β-D-glucopyranoside; Figure 3N) is a natural flavonoid present in various medicinal plants such as: *Crataegus* L*., Vigna* Savi*, Passiflora cristalina* Vanderpl. & Zappi, *Mimosa* L*.*, bamboo, etc (He et al., 2016). It has many pharmacological activities, anti-inflammatory, antiviral, anticancer, antihypertensive (Ding et al., 2021; Chen Y. et al., 2021). At the same time, Vitexin is an antioxidant (Ożarowski and Karpiński 2020). In H_2_O_2_-induced human melanocyte PIG1, Vitexin inhibited hydrogen peroxide induced apoptosis and promoted cell proliferation by activating the MAPK-Nrf2/ARE pathway, including decreasing IL-1β. The expression of IL-17A, Bax, caspase-3 and ROS, up-regulated the expression of p53, Bcl-2, Nrf2, HO-1, NQO-1, SOD (Li et al., 2020).

### Hyperoside

Hyperoside (quercetin-3-O-galactoside, Hyp; Figure 3O) belongs to flavonol glycosides isolated from *Rhododendron brachycarpum* D. Don ex g. don, *Abelmoschus manihot* (L.) Medik*. Rhododendron* L*.*(Zhou et al., 2021). Studies have shown that Hyp possesses antioxidant, anticancer, antifibrotic, antiallergic, anti-inflammatory and other pharmacological activities (Huang et al., 2020). Hyperoside markly increased the proliferation of melanocytes in a dose - and time-dependent manner *in vitro*. In the H_2_O_2_-induced melanocyte model, Hyp protected melanocytes from oxidative damage by regulating the PI3K/Akt pathway, inhibiting p38 phosphorylation and suppressing mitochondrial apoptotic signaling, which included upregulation of the Bcl-2/Bax ratio and expression of Akt, and downregulation of caspase 3, p38 (Yang et al., 2016).

### Baicalin

Baicalin (7-glucuronic acid-5,6-dihydroxy-flavone, BA; Figure 3P) is a kind of small molecular flavonoids extracted from *Scutellaria baicalensis* Georgi (Fan et al., 2021). BA has a variety of pharmacological activities, such as antioxidant stress, regulation of immunity, regulation of lipid metabolism disorders, anti-inflammatory and improve cell apoptosis (Xin et al., 2020). Recent studies have found that baicalin mediates antioxidant stress by activating Nrf2 signaling pathway (Wang X. et al., 2021). In the 40% monobenzone cream-induced vitiligo model, BA intraperitoneal injection inhibited the infiltration of leukocytes and CD8 + T cells in vitiligo lesions, increased the tyrosinase activity in the lesion area, reduced the expression of chemokine CXCL10 and its receptor CXCR3, and reduced the expression of inflammatory factors in serum samples, including IL-6 and TNF- α, IFN- γ And IL-13 (Zhu et al., 2019). The results of this study are obviously exciting. The infiltration of active CD8 + T cells occurs around the lesions of vitiligo, which is an important reason for the immune destruction of melanocytes (Wu et al., 2013). *In vivo* experiments, BA could inhibit the infiltration of immune T cells, remove inflammatory factors to slow down the appearance of leukoplakia, and reduce the area of decolorized spots. So it is a potential drug for the treatment of vitiligo.

### Polyphenol

#### Epigallocatechin-3-Gallate

**FIGURE 4:**
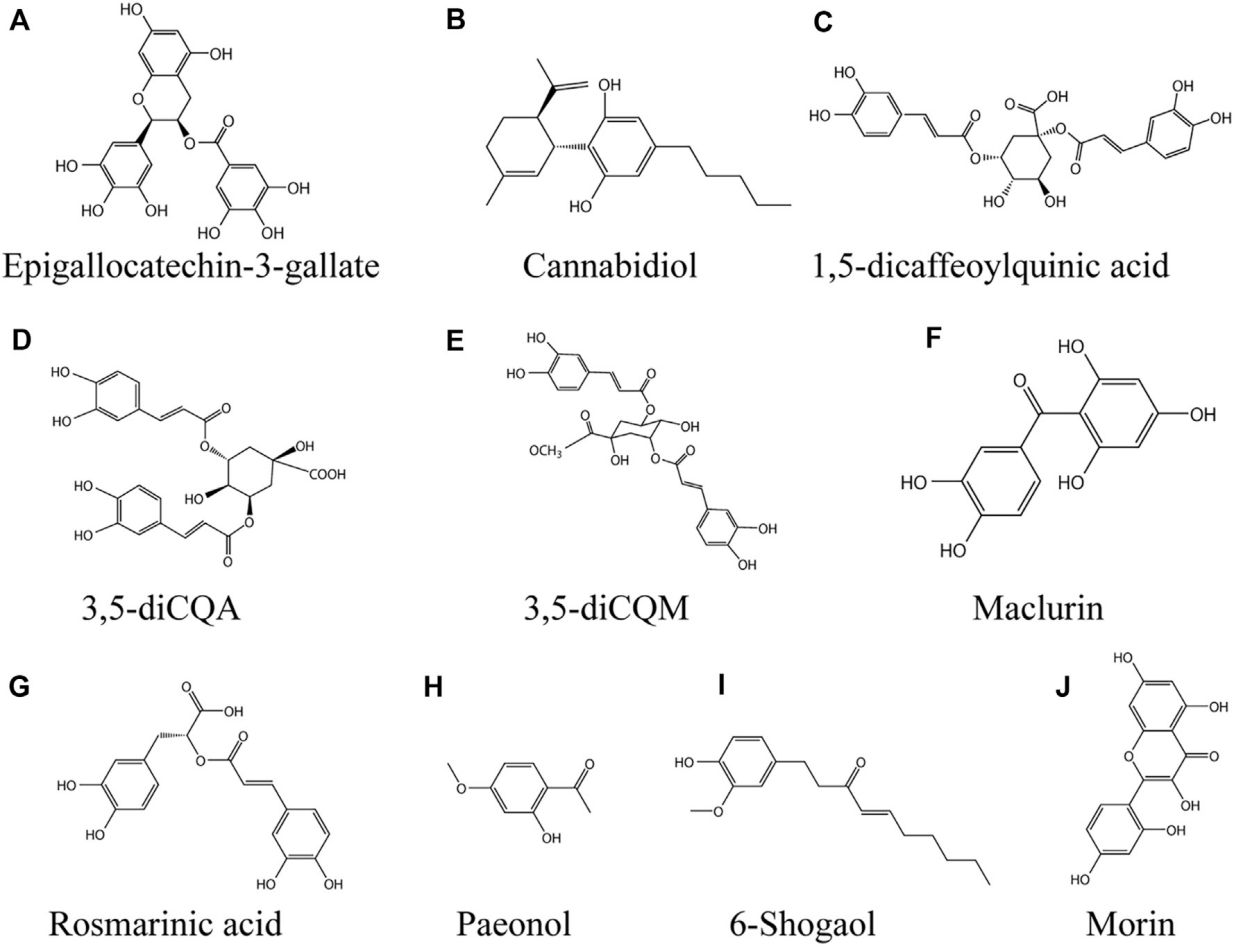
Phenolic compounds against vitiligo **(A)** EGCG, **(B)** Cannabidiol, **(C)** 1,5-dicQA, **(D)** 3,5-diCQA, **(E)** 3,5-diCQM, **(F)** Maclurin, **(G)** Rosmarinic acid, **(H)** Paeonol, **(I)** 6-Shogaol, **(J)** Morin.

Epigallocatechin-3-gallate (EGCG, Figure 4A) belongs to catechin polyphenols and is one of the main bioactive substances in Camellia sinensis (L.) Kuntze (Huang et al., 2021). It has many pharmacological effects, including anti-inflammatory, anti-atherosclerotic and anti-cancer effects (Mereles and Hunstein 2011), it's also an antioxidant (Kalaiselvi et al., 2013). Katiyar et al. suggested that EGCG could be a topical preparation to resist UVB-induced ROS-related inflammatory skin diseases, photocarcinogenesis and photoaging (Katiyar et al., 1999). In a model of monobenzone-stimulated vitiligo in mice, EGCG delayed the time to depigmentation, the area of depigmentation and reduced the incidence of hyperpigmentation in the dorsal skin of mice. The underlying mechanism was the inhibition of CD8 + T cell migration and inflammatory cytokine expression. Meanwhile, EGCG decreased the expression of IFN-γ, TNF-α, and IL-6 in serum. 5%EGCG cream is the optimal concentration for the treatment of vitiligo (Zhu et al., 2014). IFN - γ, which plays a key role in vitiligo pathogenesis, feedback through crosstalk to promote CD8 + T cell recruitment to the skin (Harris et al., 2012). High levels of CXC chemokines induced by IFN - γ such as CXCL9, CXCL10 and CXCL11 were also found in patient serum, being the most highly expressed genes in the transcriptional profile of skin lesions from vitiligo patients (Rashighi et al., 2014). Excitingly, EGCG inhibited IFN - γ - induced phosphorylation activation of JAK2, STAT1 and STAT3 in human melanocytes and significantly suppressed the levels of ICAM-1, CXCL10 and MCP-1 in a dose-dependent manner. In primary cultured human melanocytes. EGCG reduced the expression of CXCR3, CCR2, and CD11a in purified CD8 + T cells derived from the CD4 + T leukemia cell line Jurkat and peripheral blood monoclonal cells (PBMCs) in a dose-dependent character (Ning et al., 2015).

### Cannabidiol

Cannabinediol (CBD; (Figure 4B) non-psychoactive compound extracted from Cannabis sativa L., is an open pyran ring analogue. CBD has anti-inflammatory, antioxidant and anti-apoptotic effects (Iuvone et al., 2009). In addition, it has immunomodulatory properties (Mechoulam and Hanus 2002). In recent years, it has attracted great attention due to its potential in the treatment of various pathological diseases, including skin and cosmetic diseases (Baswan et al., 2020). CBD increaseed melanin content by binding to CB1 receptor of human epidermal melanocytes. The mechanism is to up regulate the expression of MITF by activating p42/44MAPK and p38MAPK signaling pathways, and then promote melanogenesis (Hwang et al., 2017). The safety of CBD transdermal drug delivery system is also under development (Paudel et al., 2010).

### 1,5-Dicaffeoylquinic Acid

1,5-dicaffeoylquinic acid (1,5-dicQA; Figure 4C) is a natural polyphenol widely found in Baccharoides anthelmintica (L.) Moench and Helianthus annuus L. (Cheevarungnapakul et al., 2019; Dogra et al., 2020). 1, 5-diCQA has a wide range of pharmacological effects, such as antioxidant, neuroprotective, antifibrotic and other biological activities (Cao et al., 2010). Plant seeds containing 1, 5-diCQA are used in traditional medicine to treat vitiligo (Tuerxuntayi et al., 2014). A previous study found that 1,5-dicqa exerts antioxidant activity through reducing intracellular ROS level and Nrf2 dependent pathway (Cao et al., 2010). 1, 5-diCQA inhibited Aβ42-induced neurotoxicity by activating PI3K/Akt, decreasing GSK3 β level and regulating Bcl-2/Bax ratio (Xiao et al., 2011). B16 cells treated with 0, 5, 50 or 100 μM 1, 5-dicQA significantly up-regulated the transcription levels of MITF, TYR, TRP1 and TRP2 in a dose-dependent manner without cytotoxicity. The mechanism is to activate p38 MAPK, ERK MAPK and PKA signaling pathway to promote the melanin synthesis of B16 cells. In conclusion, these studies suggest that 1,5-dicQA may be an effective drug for the treatment of hypopigmented skin diseases (Mamat et al., 2018).

### 3,5-diCQA

3,5-caffeoylquinic acid (3,5-diCQA; Figure 4D) is a polyphenolic compound extracted from the root of Cichorium intybus L. (Legrand et al., 2016), it also accumulated in *Achillea millefolium* L*.* and *Artemisia dracunculus* L*.* 3,5-diCQA has high free radical scavenging activity (Bernard et al., 2020). Phenolic compounds are beneficial to human health, especially their anti-inflammatory and antioxidant properties (Miguel et al., 2020). Plant polyphenols help the skin resist the damage caused by sunlight (Perez-Sanchez et al., 2016). 3,5-diCQA increased the content of melanin in melanocytes in a dose-dependent manner, the possible mechanism is that 3,5-diCQA promoted the phosphorylation of Akt and GSK-3 β, leading to the accumulation of β - Catenin in the cytoplasm. Subsequently, β - Catenin transferred to the nucleus and bound to LEF, which increased the protein expression of downstream MITF, TYR, TRP1 and TRP2. Therefore, 3,5-diCQA may restore skin pigmentation under the loss of antioxidant enzymes or melanocyte dysfunction (Mamat et al., 2017).

### 3,5-diCQM

3,5-dicaffeoylquinic acids (3,5-diCQM; Figure 4E) is a sort of natural phenolic acids condensed from quinic acid and caffeic acid by esterification. It is widely found in plants such as Gloriosa superba L.*,* Inula helenium L.*, Xanthium strumarium* subsp. strumarium*,* Crataegus azarolus L. and other fruit trees (Bernard et al., 2020). Caffeoylquinic acid derivatives have been used in traditional medicine in the east to treat a variety of diseases and show a diversity of pharmacological effects, such as liver protection (Basnet et al., 1996), anti-microbial (Zhu et al., 2004), anti-inflammatory (Góngora et al., 2002). In addition, the antioxidant activity of caffeoylquinic acid derivatives has been reported (Lin Y.-L. et al., 2011; Jang and Koh 2019). In the experiment of B16F10 melanoma cells treated with 0–50 μm 3, 5-dicCQM, the results showed that 3,5-diCQM could induce pigmentation. 3,5-diCQM drives melanogenesis in a dose-dependent manner, and the molecular mechanism underlying its ability to induce pigmentation is through activation of the p38 signaling pathway, phosphorylation and activation of CREB, and a cAMP/PKA dependent signaling pathway, which in turn upregulates the transcription factor MITF, thereby activating tyrosinase activity (Kim et al., 2015).

### Maclurin

Maclurin [(3,4-dihydroxyphenyl) -(2,4,6-trihydroxyphenyl; Figure 4F) methanone] is a natural phenolic compound that belongs to the benzophenone family and is found in *Morus alba* L*., Garcinia mangostana* L. Previous studies have found that maclurin has pharmacological activities such as anticancer, antioxidant and anti-skin aging (Lee 2018; Lee et al., 2018; Mi Moon et al., 2019). The content of melanin in melanocytes was increased by maclurin in a dose-dependent manner, by a mechanism involving the activation of the cAMP/PKA/CREB signaling pathway, which in turn increased tyrosinase, MITF and their downstream TRP-1 and Trp-2 protein content, while also involving the activation of p38 MAPK and p44/42 MAPK pathways. *In vitro*, maclurin significantly attenuated UVB induced ROS production, inhibited hydrogen peroxide induced reduction of melanin and decreased cell survival (Hwang et al., 2019).

### Rosmarinic Acid

Rosmarinic acid (a-o-caffeoyl-3,4-dihydroxyphenyl lacticacid; Figure 4G) is a natural phenolic compound, which exists in many Labiatae plants, such as *Perilla L.*, *Rosmarinus officinalis* L*.*, *Prunella vulgaris* L. (Zhang et al., 2021). Rosmarinic acid has anti-inflammatory, anti-oxidation, anti-cancer and other pharmacological activities (Wang L. et al., 2019; Zhang et al., 2021). Incubation of 50 μm rosmarinic acid with B16 melanoma cells for 48 h resulted in a significant increase in melanin content and tyrosinase protein expression, the mechanism being that rosmarinic acid induces melanin synthesis by activating the PKA/CREB signaling pathway via phosphorylation (Shomirzoeva et al., 2019). Recently, ultradeformable liposomes (UL) have been developed by scientific researchers, which greatly increased the skin permeation ability of rosmarinic acid by UL containing oleic acid, exhibiting potential as a formulation for development for external use (Subongkot et al., 2021).

### Paeonol

Paeonol (Pae; 2′-hydroxy-4-methoxyacetophenone; Figure 4H) is a natural phenolic compound extracted from the *Paeonia* × *suffruticosa* Andrews (Miao et al., 2021). Paeonol has been used in traditional Chinese medicine as an anti-inflammatory and antipyretic drug with cardiovascular, anti-inflammatory, neuroprotective, antitumour and other pharmacological activities (Zhang L. et al., 2019; Tsai et al., 2020). Paeonol alleviated UVB-induced skin photoaging by activating Nrf2 and the antioxidant response element (Sun et al., 2018). Paeonol exhibits anti-inflammatory, anti-allergic activity in animal models of atopic dermatitis and psoriasis (Meng et al., 2017; Meng et al., 2019). In H_2_O_2-_induced PIG1 oxidative stress model of normal human epidermal melanocytes, paeonol inhibited hydrogen peroxide induced decrease in cell viability in a dose-dependent manner. 20 μM paeonol increased the enzymatic activities of SOD, CAT, GSH-Px and the expression of HO-1, NQO1, and SOD2 by activating the Nrf2 signaling pathway, but paeonol was unable to affect melanogenesis in PIG1 cells and acted only as a protective agent (Guo and Zhang 2021).

### 6-Shogaol

6-shogaol (Figure 4I), a natural phenolic compound, is the major active ingredient in *Zingiber officinale* Roscoe. Studies have found that 6-shogao possesses antiemetic, anti-inflammatory, antioxidant, and anticancer activities, as well as cardiovascular and neuroprotective effects (Kou et al., 2018; Chen et al., 2020; Yadav and Jang 2020). Jin et al. found that 6-shogaol exerts cellular antioxidant activity by mediating Nrf2 signaling through activation of the JNK pathway (Kim and Jang 2016). Feng et al. found that 6-shogaol inhibited UVB induced inflammation and oxidative stress in keratinocytes (Chen F. et al., 2019). Pretreatment of HEMn-MPs with 5 µM 6-shogaol for 6 h protected melanocytes from rhododendrol-induced cytotoxicity and maintained their original cell viability. In the oxidative stress-induced HEMn-MPs model, cells pretreated with 6-shogao maintained the initial cellular morphology, and 6-shogao significantly attenuated H_2_O_2_ induced oxidative stress and death and melanogenesis inhibition in melanocytes (Yang L. et al., 2020).

### Morin

Morin (2′,3,4′,5,7-pentahydroxyflavone; Figure 4J) is a natural polyphenol found in onion, fig, guava leaves, apple and other Moraceae families such as *Psidium guajava* L.*, Maclura pomifera* (Raf.) C.K.Schneid., as well as the medicinal plant *Alpinia officinarum* Hance (Lotito and Frei 2006). Studies have found that Morin has antitumor, antihypertensive, antioxidant, anti-inflammatory, antidiabetic, neuroprotective, antibacterial and other pharmacological effects (Caselli et al., 2016; Heeba et al., 2021). 50 μM Morin significantly upregulated the expression of MITF, as well as its downstream TRP-1 and Trp-2 to increase melanin production in B16F10 mouse melanoma cells, and the mechanisms include the activation of ERK and p38 signaling pathways via the phosphorylated MAPK pathway (Shin et al., 2021). Excitingly, long-term doses of oral Morin did not exhibit any toxicity (Cho et al., 2006).

## Glycosides

### Geniposide

**FIGURE 5:**
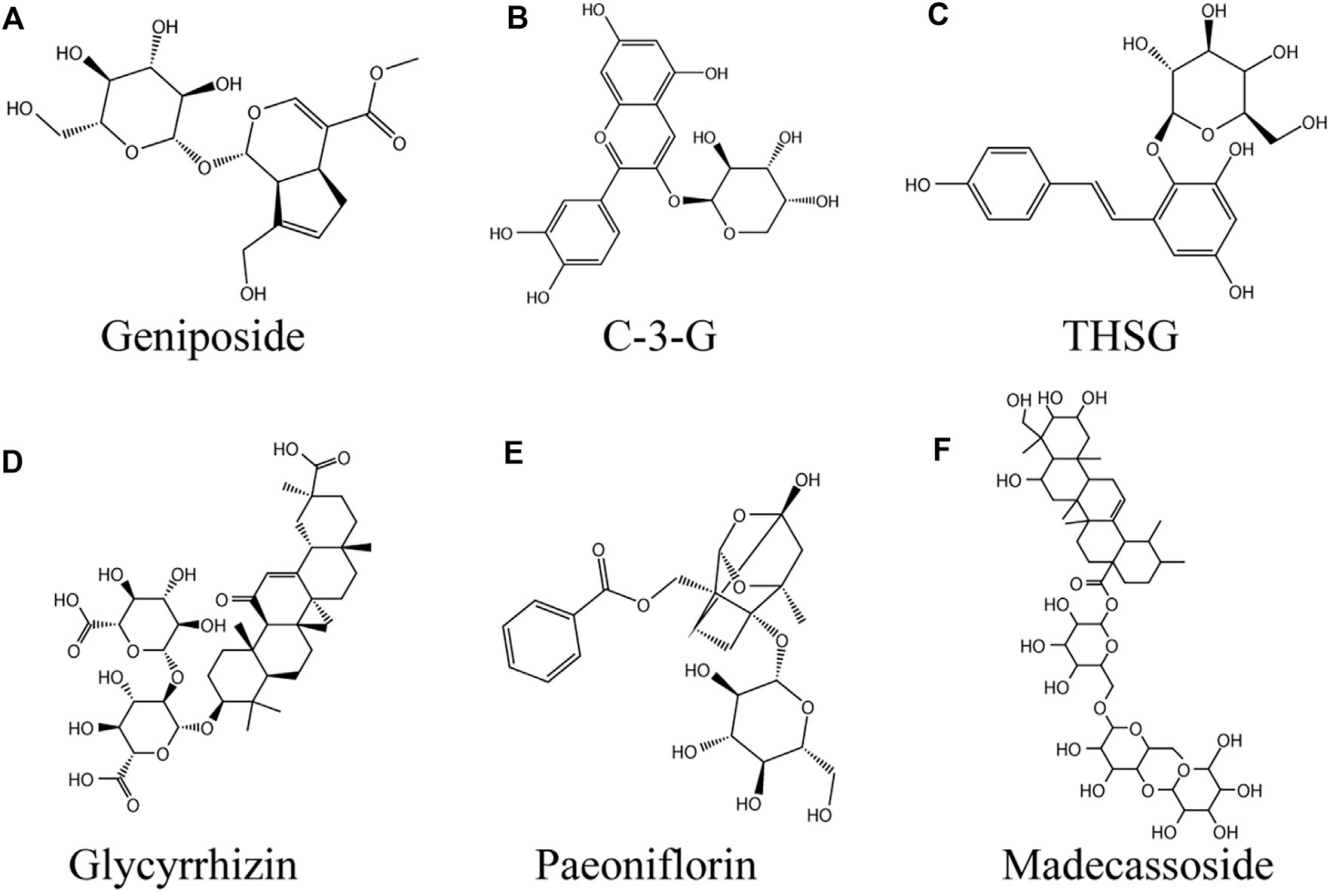
Glycosides against vitiligo **(A)** Geniposide, **(B)** C-3-G, **(C)** THSG, **(D)** Glycyrrhizin, **(E)** Paeoniflorin, **(F)** Madecassoside.

Geniposide (GP; Figure 5A) (C17H24O10) is a sort of iridoid glycoside extracted from Gardenia jasminoides J. Ellis fruits, which widely exists in nearly 40 species of plants in various families, especially Rubiaceae (Shan et al., 2017). In terms of biological activity, GP has been found to have a variety of pharmacological effects, such as anti-diabetes (Zhang et al., 2016), neuroprotective (Zhao et al., 2017), anti-inflammatory, antioxidant, etc (Zhou et al., 2019). GP is also an important component in many traditional Chinese herbal medicines for the treatment of vitiligo, such as Eucommia ulmoides Oliv. and Rehmannia glutinosa (Gaertn.) DC. (Zhou et al., 2019). In HEMn or HEKn models induced by norepinephrine (NE), GP significantly abolished the inhibitory effect of NE on HEMn melanogenesis in the presence of recombinant SCF. The binding of NE to α 1-adrenoceptor in melanocytes decreased cAMP level, resulting in decreased intracellular calcium uptake associated with c-kit production. The mechanism of GP promoting melanogenesis was through activating GLP-1R/c-kit receptor signal to enhance the expression of c-kit receptor, so as to eliminate the depigmentation caused by norepinephrine (Wen-Jun et al., 2008). In the melanocytes induced by H_2_O_2_, GP increased the activities of SOD and CAT, reduced the accumulation of ROS, thus increased the antioxidant capacity of melanocytes and inhibited melanocyte apoptosis by upregulating the Bcl-2/Bax ratio, while increasing cell viability. This process involves activation of PI3K/Akt signaling pathway (Zhou et al., 2019).

### Cyanidin-3-o-β-Glucopyranoside

Cyanidin-3-o-β-glucopyranoside (C-3-G; Figure 5B) is a kind of anthocyanin flavonoids widely found in vegetables and fruits. Chinese *Myrica cerifera* L. is a rich source of C-3-G, which is one of the most common anthocyanins (Sun et al., 2013). Anthocyanins are beneficial to human body. Known pharmacological actions include anti-inflammatory, anti-fatigue, anti-oxidation, anti-tumor and so on (Cui et al., 2018). In addition to the ability to significantly reduce ROS mediated oxidative damage, C-3-G inhibited UVA induced damage to primary human dermal fibroblasts (HDFS) by inducing autophagy (Wu et al., 2019). In one study, TVM-A12 human melanoma cells were incubated with 5 μm C-3-G, and the cells return to the differentiation state from the proliferation state. In addition, two human melanoma cell lines A-375 and M14 also obtained dendritic phenotypes after C-3-G treatment. At the concentration used, the cells treated with C-3-G showed no apoptosis or necrosis. At the same time, C-3-G significantly up-regulated the expression of tyrosinase, thereby inducing the content of melanin in TVM-A12 cells, which was mediated by up regulating cAMP pathway. It is particularly encouraging that the active concentration of C-3-G is comparable to food intake (range of μm) and has no toxicity. (Serafino et al., 2004).

### 2, 3, 5, 4′-Tetrahydroxystilbene-2-O-β-D-Glucoside

2, 3, 5, 4′-tetrahydroxystilbene-2-O-β-D-glucoside (THSG; Figure 5C) is a water-soluble active ingredient in Reynoutria multiflora (Thunb.) Moldenke (He et al., 2015). Numerous studies have found that THSG exerts antioxidant effects by protecting cells from oxidative damage caused by H_2_O_2_ through increasing SOD activity, reducing MDA content, and inhibiting ROS production (Yang et al., 2014; Wu et al., 2020). In hairless skin model, the extract of *Reynoutria multiflora* could up regulate SOD in a dose-dependent manner, thereby reducing UVB-induced oxidative damage, suggesting that the extract of *Reynoutria multiflora* contains anti skin photoaging agent (Hwang et al., 2006). At the first time, it was proved to be an effective tyramine Acid enzyme activators and melanogenesis stimulants (Guan et al., 2008). THSG increased tyrosinase activity in a dose-dependent manner at concentrations ranging from 1 to 10 μg/ml (Jiang et al., 2009). Guan et al. (2008) reached a similar conclusion. The mechanism was that THSG directly activated AC or inhibited PDE, increased cAMP level in cytoplasm, and mediated the activation of MITF/CREB. P38MAPK had a regulatory effect on melanin formation and the induced expression of tyrosinase and MITF in B16 cells induced by THSG (Jiang et al., 2009).

### Glycyrrhizin

Glycyrrhizin (GLC; (Figure 5D) natural triterpene saponin, is one of the major chemical components extracted from *Glycyrrhiza* Tourn. ex L*.* and has been widely used in Asia, Europe, the Middle East (Cai et al., 2017). Studies have shown that GLC has antiallergic, immunomodulatory, antiulcer, anticancer, antioxidant, and antiviral effects (Wang G. et al., 2021). Jung et al. were the first to find that GLC induced melanin production in B16 melanoma cells in a dose-dependent manner by upregulating the tyrosinase and Trp-2 genes (Jung et al., 2001). Li et al. also verified this conclusion. Further studies revealed that GLC increases melanin production in melanocytes by three pathways, 1) activating activator protein-1 (AP-1) and CRE promoter to activate p42/44 MAPK signaling, 2) activating cAMP signaling, and 3) decreasing GSK3β phosphorylation while inducing CREB phosphorylation (Lee et al., 2005). Pretreatment with 1 mM GLC significantly inhibited H_2_O_2_-induced melanocyte apoptosis, and GR protected melanocytes from oxidative stress by reducing ROS production in cells via activation of the Nrf2/HO-1 pathway (Mou et al., 2019). Clinical study observations suggest that treatment with oral GLC in combination with UVB improves active generalized vitiligo (Mou et al., 2016).

### Paeoniflorin

Paeoniflorin (C_23_H_28_O_11_, PF; (Figure 5E) monoterpene glycoside, is a major active ingredient isolated from the roots of *Paeonia veitchii* Lynch or *Paeonia lactiflora* Pall., which has been applied for 1,200 years in China (Li J. et al., 2021). *In vivo* and *in vitro*, paeoniflorin has a wide range of pharmacological activities, including anti-inflammatory, antioxidant, immunomodulatory, analgesic, anticonvulsant, antithrombotic, neuroprotective, cardioprotective, hepatoprotective, antitumor and antidepressant effects (Zhou Y.-X. et al., 2020). In the 40% monobenzone-induced vitiligo mouse model, the number of hair follicles and melanin content in the skin of paeoniflorin treated for 10 days were significantly higher than those of the model group. *In vitro*, 10 μg/ml paeoniflorin can stimulate the synthesis of melanin, and its mechanism is to increase the protein level of MITF and its downstream TRP-1 by promoting the phosphorylation of ERK and CREB (Hu M. et al., 2020). Interestingly, PF protected H_2_O_2_-induced PIG1 and PIG3V cells from oxidative stress by activating JNK/Nrf2/HO-1 signaling pathway, including decreased ROS level and increased SOD, CAT, Nrf2 and HO-1 expression. PF at a concentration of 50 μM, the cell viability was significantly increased (Yuan et al., 2020).

### Cistanche deserticola Polysaccharide

Cistanche deserticola polysaccharide (CDP) is the main active component isolated from *Cistanche deserticola* Ma. It is widely used in anti-virus and anti-tumor in North Africa, Arab and Asian countries (Gu et al., 2016). In addition, CDP also has the pharmacological activities of liver injury protection, lipid balance, anti-aging, regulating immune function and antioxidant (Liu et al., 2018). CDP promoted the formation of melanin *in vivo* and *in vitro*. CDP promoted melanogenesis by activating MAPK signaling pathway and up regulating the expression of MITF and its downstream genes Tyr, TRP1 and Trp2, including increasing the phosphorylation levels of p38, JNK and ERK proteins. *In vivo*, CDP promotes melanin production in zebrafish. Notably, CDP protected HEM and B16F10 cells from oxidative stress induced by H_2_O_2_ and significantly inhibited apoptosis induced by oxidative stress (Hu Y. et al., 2020).

### Madecassoside

Madecassoside (MADE; (Figure 5F) natural triterpenoid saponin, is isolated from *Centella asiatica* (L.) Urb (Zhou J. et al., 2020). Studies have found that asiatic acid has a wide range of pharmacological activities, such as anti-apoptotic, anti-inflammatory, and anti-oxidative (Peng et al., 2020). In the H_2_O_2_-induced oxidative stress model, MADE reduced the shrinkage of dendrites of melanocytes affected by oxidative stress in a dose-dependent manner, maintained cell morphology, improved mitochondrial swelling, and exerted antioxidant activity by increasing autophagy by upregulating the levels of LC3-II and LC3-I in melanocytes. Therefore, MADE is a promising natural product against vitiligo (Ling et al., 2017).

### Coumarin

#### Psoralidin

**FIGURE 6:**
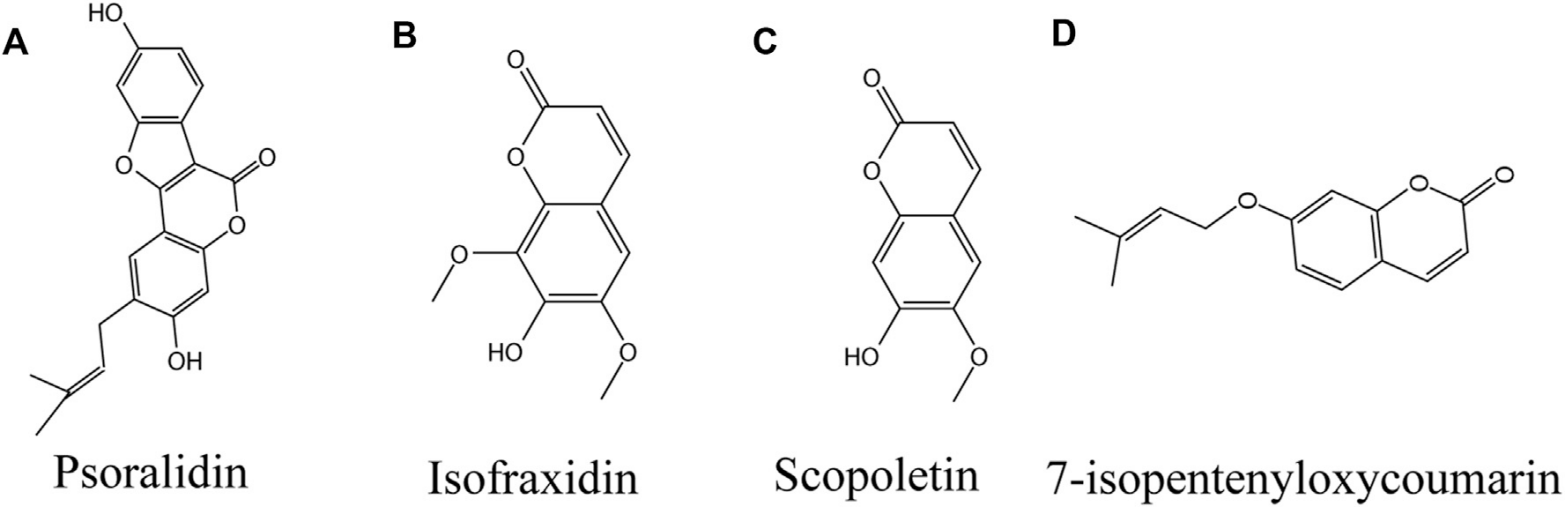
Coumarins against vitiligo **(A)** Psoralidin, **(B)** Isofraxidin, **(C)** Scopoletin, **(D)** 7-isopentenyloxycoumarin.

Psoralidin (PL; Figure 6A), a natural coumarin isolated from *Cullen corylifolium* (L.) Medik. seeds, is structurally similar to coumestrol (Cao et al., 2019). It also exists naturally in various plants, such as lemon, lime and parsnip divaricata. PL is beneficial to diabetic complications, oxidative stress, obesity, osteoporosis, apoptosis, autophagy and cell proliferation (Sharifi-Rad et al., 2020). PL is used in the traditional Uyghur medicinal materials for color restoration (Wang et al., 2014; Niu et al., 2016; Pei et al., 2016), and several psoralen compounds, such as 8-MOP and 5-MOP, isolated from the same plant (Zang et al., 2019), are used in ultraviolet color restoration therapy (Dincer Rota et al., 2021). *In vitro* experiments showed that PL could improve the activity of tyrosinase (Shi et al., 2018). A recent clinical controlled study found that treatment of vitiligo lesions with psoralen in combination with NBUVB had higher efficacy compared with NBUVB alone, and there were no serious complications (Zabolinejad et al., 2020). The mechanism may provide an antioxidant effect by regulating the PI3K/Akt signaling pathway, affecting the downstream GSK3 β/β - Catenin, and the Nrf2/HO-1 axis (Zhai et al., 2018).

### Isofraxidin

Isofraxidin (Figure 6B) is a natural coumarin isolated from Artemisia capillaris Thunb. (Yim et al., 2017b). Isofraxidin related derivatives have been found to have anti-inflammatory, antioxidant, neuroprotective and other biological activities (Lau et al., 2019; Majnooni et al., 2020). Yim et al. emphasized that isofraxidin may be a new type of pigmentation agent. At the dose of 12.5 μm and 25 μm, zebrafish treated with isofraxidin showed higher melanin synthesis and tyrosinase activity *in vivo* experiments. *In vitro*, 25 μm isofraxidin significantly increased the melanin content in B16F10 cells by up regulating MITF and tyrosinase gene expression (Yim et al., 2017a).

### Scopoletin

Scopoletin (SP; Figure 6C) is a phenolic coumarin isolated from Evolvulus alsinoides (L.) L., which has many pharmacological effects (Zhang F. et al., 2019). It has been isolated from Gramineae, Liliaceae, Musaceae, Compositae, Convolvulaceae and Leguminosae (Tal and Robeson 1986; Gnonlonfin et al., 2011). SP has been reported to have anti-inflammatory effect (Chaingam et al., 2020) and antioxidant effect (Usman et al., 2020), When B16F10 melanoma cells were treated with 0–50 μm SP, it was found that SP to induce melanin synthesis in a dose-dependent manner by increasing the expression of MITF and tyrosinase via increasing CREB phosphorylation (Ahn et al., 2014). When zebrafish embryos were exposed to the compound for 2 days, the increase of pigment was detected. *In vitro*, SP at concentrations of 0–50 μmol/L enhanced melanogenesis *in vivo* and *in vitro* by increasing melanin content and Tyr and MITF expression (Heriniaina et al., 2018).

### 7-Isopentenyloxycoumarin

7-isopentenoxycoumarin (Figure 6D), a natural pentenoxyumbelliferone derivative widely found in Rutaceae and umbelliferaceae, is a floral extract of *Amaranthus retroflexus* L., which is used as a food product in southern and central Italy (Fiorito et al., 2017). Studies have shown that 7-isopentenyloxycoumarins have antifungal, antioxidant, anticancer, neuroprotective, and anti-inflammatory properties (Preziuso et al., 2020). Fiorito et al. found that 7-isopentenoxycoumarin significantly induced melanogenesis at a dose of 40 μm, with the highest induction of melanin at 72 h, and excitingly the induction was 6-fold greater than that of the control group, and the mechanism of action was to increase melanogenesis by elevating MITF and its downstream genes tyrosinases, TRP-1 and Trp-2. Interestingly, 7-isopentenoxycoumarin interacted with the ERβ (ER-β) Binding may also be involved in melanin biosynthesis, as this receptor antagonist inhibited melanogenesis (Fiorito et al., 2018).

## Other Compounds

### Sesamin

**FIGURE 7:**
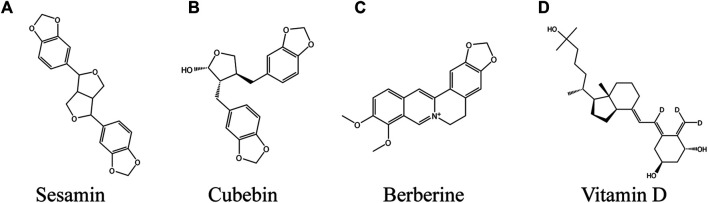
Coumarins against vitiligo **(A)** Sesamin, **(B)** Cubebin, **(C)** Berberine, **(D)** Vitamin D.

Sesamin (Figure 7A), a kind of lignan found in oil and Sesamum indicum L. seed, has been found to have a variety of biological activities and is beneficial to human body (Udomruk et al., 2020). The high antioxidant activity of sesamin has been reported (Pathak et al., 2014). In addition, its anti-nociceptive and anti-inflammatory activity was also reported (Jeng et al., 2005; Monteiro et al., 2014). It increased the content of tocotrienoll in the skin, so as to reduce sunburn and tumor incidence rate (Yamada et al., 2008), reduced the skin erythema caused by UVB, improved skin inflammation, protected skin from wrinkle formation and light damage (Lin et al., 2019). In the concentration range of 1–10 μM, sesamin increased melanin production in a dose-dependent manner. The mechanism is that sesamin up regulated CREB gene by activating cAMP/PKA signaling pathway, then up regulated the expression of tyrosinase and MITF, and finally induced melanin synthesis (Jiang et al., 2011). The currently developed transdermal drug delivery system will successfully transform lignin into an external preparation for the treatment of vitiligo in the future (Nguyen et al., 2015).

**TABLE 1:** Summary of natural products for the treatment of vitiligo in models.

	Natural compounds name	Models	Range of dosage	Targets/pathway/process/mechanism	References
Flavonoids	Baicalein	H_2_O_2_-induced PIG3V (human cell)	50 µM	Cell vitality and proliferation, Nrf2, HO-1, SOD2, NQO1, ERK1/2, PI3K/AKT	Ma et al. (2018)
		H_2_O_2_-induced PIG1 (human cell)	10, 20, and 40 µM	Cell viability, ROS, Bax, Bcl-2, caspase-3, β-actin, COX4, Cyt-4, p38 MAPK, ERK	Liu et al. (2012)
	Quercetin	H_2_O_2_-induced epidermal melanocytes (human cell)	25 µM	TYRe, Cell viability, ROS production, β-actin	Guan et al. (2015)
	Kaempferol	HMVII (human cell)	1, 5, 10 and 20 µM	TYR activity, melanin content	Takekoshi et al. (2014)
		B16F10	8, 16, 32 μM	Viability, melanin melanin contents, Synthesis, TYR activity, MC1R, MITF, TYRP1, DCT	Wang et al. (2017)
	Apigenin	H_2_O_2_-induced PIG3V (human cell)	1, 5, 10 and 20 μM	Cell viability, SOD, CAT, GSH-PX, MDA, Nrf2, HO-1, NQO1, β-actin	Zhang et al. (2020)
		Dopamine-induced melanocytes (human cell)	0.3–10 μM	Cell viability, ROS, JNK, p38MAPK, Akt, caspase-3, PARP	Lin et al. (2011a)
	Galangin	Male C57BL/6 (mice)	0.425 mg/kg, 4.25 m g/kg	Melanocytes, TYR activity, CHE activity, MDA	Huo et al. (2014)
	Naringenin	B16 melanocytes 4A5 (mice cell)	0, 10, 25, 50, 100, 200 μM	Tyrp1, Dct, MITF, ERK, PI3K, GSK-3β, β-actin	Ohguchi et al. (2006)
		B16-F10 (mice cell)	3–50 μM	Cell viability, melanin content, TYR, MITF, β-catenin, GSK-3β, Akt	Huang et al. (2011)
	Hesperetin	B16-F10 (mice cell)	3, 10, 30, 50 μM	Cell viability, melanin content, TYR, MITF, β-catenin, p38 MAPK, GSK-3β, p38MAPK, ERK, Akt, CREB	Huang et al. (2012)
	Afzelin	Epidermal melanocytes (human cell)	0, 10, 50, 100 μM	TYR activity, Cell viability, MITF, TRP-1, TRP-2, p42/44MAPK, p38MAPK, JNK, CREB	Jung et al. (2016)
	Fisetin	B16F10 (mice cell), zebrafish model	5, 20 µM	MITF, TYR, cell viability, melanin Content	Molagoda et al. (2020)
	Puerarin	Melan-a cell, zebrafish model, MITFvit/vit mice	50 µM	MITF-M, Bcl-2, TRP-2, TYR, cAMP, melanin content cell viability, melanin content, TYR, TRP-1, MITF, GAPDH, ERK1/2, p38, JNK	Park et al. (2014)
		40% monobenzone cream-induced emale C57BL/6 (mice), melanocytes from foreskins (human cell)	1, 5, 10, 20, 40 μmol/L		Ding et al. (2019)
	Butin	Hydroquinone-induced mice	0.425, 4.25, 42.5 mg/kg	TYR, Trp-1, CHE, MDA	Huo et al. (2017)
		B16 cell, H_2_O_2_-induced zebrafish	1, 10, 100 µM	ROS, melanin Content, TYR	Lai et al. (2021)
	Liquiritin	B16-F1 (mice cell), HMVII (human cell)	12.5, 25, 50 µM	Cell viability, melanin Content, TYR, TRP-1, TRP-2, p38, CREB, MITF, GAPDH	Uto et al. (2019)
	Liquiritigenin	B16-F1 (mice cell), HMVII (human cell)	12.5, 25, 50 µM	Cell viability, melanin Content, TYR, TRP-1, TRP-2, p38, CREB, MITF, GAPDH	Uto et al. (2019)
	Vitexin	H_2_O_2_-induced PIG1	10, 20, 30, 40 µM	Cells viability, IL-1β, IL-17A, Nrf2, HO-1, NQO-1, SOD, cyt-C, ERK, ROS, p53, Bax, Bcl-2, β-actin, caspase-3	Li et al. (2020)
	Hyperoside	H_2_O_2_-induced epidermal melanocytes (human cell)	2, 10, 50 µg/ml	Cell viability, melanin amount, Bcl-2, Bax, caspase 3, GAPDH, AKT, p38	Yang et al. (2016)
	Baicalin	40% monobenzone-induced C57BL/6 (mice)	20 mg/ml	CD8+T, CXCL10, CXCR3, IL-6, TNF-α, IFN-γ, IL-13	Zhu et al. (2019)
Polyphenol	EGCG	IFN-γ-induced (human cell), CD8+T	0, 10, 20, 40 µM	ICAM-1, CXCL10, and MCP-1, JAK2/STATs, CD11a, CXCR3, CCR2	Ning et al. (2015)
		Female C57BL/6 (mice)	2%, 5% and 10% EGCG cream	CD8^+^ T cells, TNF-α, IFN-γ, and IL-6, CXCl3, CXCL5, CXCR4, S100B, TGFBR2, c-fos, Rab27A, EGFR, PI3K	Zhu et al. (2014)
	Cannabidiol	Epidermal melanocytes (human cell)	1, 3, 6 µM	MITF, TYR, TRP-1, TRP-2, p38MAPK, p42/44 MAPK, TYR activity, cell viability	Hwang et al. (2017)
	1,5-dicQA	B16 (mice cell)	0–400 µM	Tyr activity, MITF, TYR, TRP-1, TRP-2, ERK, JNK, p38, PKA	Mamat et al. (2018)
	3,5-diCQA	B16 (mice cell)	0–200 µM	TYR activity, TYR, TRP1, TRP2, MITF, β-catenin	Mamat et al. (2017)
	3,5-diCQM	B16F10 (mice cell)	0–50 µM	TYR activity, melanin content, TRP-1, TRP-2, MITF, cAMP, ERK, p38MAPK, JNK, AKT	Kim et al. (2015)
	Maclurin	H_2_O_2_-induced epidermal melanocytes (human cell)	1, 10, 50 µM	TRP-1, TRP-2, MITF, tyrosinase, cAMP, PKA, CREB, p38 MAPK, p44/42 MAPK, PKA	Hwang et al. (2019)
	Rosmarinic acid	B16 (mice cell)	1, 10, 50, 100 µM	Melanin content, CREB, p38mapk, Akt, tyrosinase	Lee et al. (2007)
	Paeonol	H_2_O_2_-induced PIG1 (human cell)	5, 10, 20, 40 µM	Cell viability, TYR, TRP-1, MITF, CAT, HO-1, NQO1, SOD2, Nrf2, GAPDH, SOD, GSH-Px	Guo and Zhang (2021)
	6-Shogaol	H_2_O_2_-induced HEMn-MPs (human cell)	5 µM	Cell viability, MITF, HO-1, NQO1, Nrf2, GAPDH	Yang et al. (2020b)
	Morin	B16F10 (mice cell)	25, 50, 100, 250, 500 μM	MITF, TRP-1, TRP-2, GAPDH, ERK, p38	Shin et al. (2021)
Glycosides	Geniposide	NE-induced HEMn (human cell)	0–100 µM	TYR activity, melanin, c-kit receptor, SCF ligand	Wen-Jun et al. (2008)
		H_2_O_2_-induced primary melanocytes (human cell)		ROS, SOD, CAT, Akt, Bcl-2, Bax, caspase 3, caspase 9, β-actin	Lu et al. (2018)
	C-3-G	TVM-A12,M14,A-375 (human cell)	5, 10 μM	Cell viability, proliferation, NF-68 kDa, NF-160 kDa, NF-200 kDa, Melan-A/MART-1	Serafino et al. (2004)
	THSG	B16F1 (mice cell)	1–10 μg/ml	Melanin amount, TYR activity, MITF, ERK, p38 MAPK, JNK, cAMP	Jiang et al. (2009)
		B16 (mice cell)	0.1–25 μg/ml	TYR activity, melanin content	Guan et al. (2008)
	Glycyrrhizin	B16 (mice cell)	0.2, 0.5, 1.0, 1.5 mM	Cell viability, melanin amount, AP-1, CREB, p42/44 MAPK, cAMP, GSK-3β, MITF	Lee et al. (2005)
		H_2_O_2_-induced NHEM (human cell)	1 mM	Cell viability, ROS, β-actin, Nrf2, HO-1, GAPDH, NQO-1, GCLC, GCLM	Mou et al. (2019)
	Paeoniflorin	Epidermal melanocytes (human cell), 40% monobenzone-inducedC57BL/6 (mice)	0, 5, 10 μg/ml, 60 mg/kg in 20% propanediol	Cell vitality and proliferation, melanin amount, TRP-1, MITF, TYR, ERK, CREB, GAPDH cell viability, ROS, SOD, CAT, Nrf2, NQO1, HO-1	Hu et al. (2020a)
		H_2_O_2_-induced PIG1, PIG3V (human cell)	50–400 µM		Yuan et al. (2020)
	*Cistanche deserticola* polysaccharide	H_2_O_2_-induced B16F10 (mice cell) and epidermal melanocytes (human cell), zebrafish	20,40,80 μg/ml	Cell viability, melanin amount, TYR, ROS, MITF, TRP1, TRP2, RAB2 7A, FSCN1, GAPDH, ERK, JNK, p38, ROS, Nrf2, HO-1	Hu et al. (2020b)
	Madecassoside	H_2_O_2_-induced B16F10 (mice cell), HEM (human cell), zebrafish	20, 40, 80 µg/ml	Cell viability, melanin amount, TYR, MITF, TRP1, TRP2, RAB2 7A, FSCN1, GAPDH, ERK, JNK, p38, ROS, Nrf2, HO-1, MAPK	Ling et al. (2017)
Coumarin	Psoralidin	In silico (no cell)	0.125, 0.25, 0.5, 1 g/ml	TYR, a rate-limiting enzyme of melanogenesis	Shi et al. (2018)
	isofraxidin	B16F10 (mice cell), zebrafish	12.5, 25 µM	Melanocytes, TYR activity, melanin, MIFT, TRP-1	Yim et al. (2017a)
	Scopoletin	B16F10 (mice cell)	0–50 μM	TYR activity, melanin content, TYR, MITF, CREB, β-actin, melanin content, TYR activity, melanin content, Cell viability, ROS	Ahn et al. (2014), Heriniaina et al. (2018)
		B16F10 (mice cell), zebrafish	10–25 μmol/L		
	7-Isopentenyloxycoumarin	Melan-a (mice cell)	1, 10, 20, 40 µM	Cells viability, melanin content, tyrosinase, TRP-1, TRP-2, and MITF	Fiorito et al. (2018)
Other compounds	Sesamin	B16F1 (mice cell)	5, 10, 20 µM	Melanin amount, TYR activity, TYR, CREB, MITF, p38MAPK, PKA, cAMP	Heriniaina et al. (2018)
	Cubebin	B16 (mice cell)	0–20 µM	Melanin amount, cell proliferation, TYR activity, p38 MAPK, ERK1/2, p70 S6K1	Hirata et al. (2007)
	Berberine	H_2_O_2_-induced PIG1 (human cell)	0.1, 1.0, 5.0 μM	Cells viability, Bax, Bcl-2, PARP, HO-1, NQO1, SOD, Nrf2, Mitf, TYR, TYRP1, DCT, IL-6, IL-8, p65, ROS	Jiang et al. (2019)
	Vitamin D	H_2_O_2_-induced PIG1, PIG3V (human cell)	1 nM	Cells viability, SOD, ROS, β-catenin, CDH3, GSK3β, Nrf2, MITF, caspase3, MDA, GAPDH, HO-1	Tang et al. (2018)

### Cubebin

Cubebin (Figure 7B) is a compound extracted from the seeds of Piper cubeba L. f. (Godoy de Lima et al., 2018). It exhibits various pharmacological activities, such as trypanosomiasis, anti-Mycobacterium (Silva et al., 2007), analgesic, anti-inflammatory (Souza et al., 2004) and vasodilator (Somani et al., 2017). *In vitro*, cubebin showed a concentration time-dependent melanogenesis activity in B16 cells. The mechanism is to promote melanin synthesis by increasing the phosphorylation level of p38 MAPK, which in turn increases the expression of MITF and tyrosinase (Hirata et al., 2007).

### Berberine

Berberine (BBR, (Figure 7C) natural isoquinoline alkaloid, is a major compound isolated from the Chinese herb *Coptis chinensis* Franch. (Wang et al., 2021d), and studies have found that BBR has pharmacological activities against cancer, hypolipidemic, cardiovascular, anti-inflammatory, and antioxidant stress (Zhao et al., 2021). Wei et al. studied the potential medicinal value of berberine in vitiligo. At 0.1–5.0 μM BBR induced melanocyte proliferation in a time-dependent manner. At the same time, BBR inhibited the oxidative damage induced by H_2_O_2_ by down regulating the activities of CAT and SOD and reducing the accumulation of ROS in PIG1 cells, 5 μM BBR inhibited the cleavage of PARP and the apoptosis of melanocytes induced by H_2_O_2_ by down regulating the ratio of Bax/Bcl-2. The antioxidant effect of BBR depends on Nrf2-ARE pathway, and the process involves up regulation of HO-1, SOD and NQO-1 protein expression, and the protective effect is obviously reduced after Nrf2 gene is knocked out. In addition, BBR enhanced the expression of MITF in melanocytes induced by oxidative stress, thereby increasing the production of melanin. Finally, BBR inhibited H_2_O_2_-induced upstream NF-κB activation and expression of IL-6 and IL-8 (Jiang et al., 2019). BBR could protect melanocytes from oxidative stress through anti-oxidation and anti-inflammatory. It is a potential natural drug against vitiligo.

### Vitamin D

Vitamin D (1,25-dihy-droxyvitamin D3, Figure 7D) is a cyclopentane phenanthrene compound, which is an essential vitamin for human body, it mainly comes from daily meat (Dominguez et al., 2021). Vitamin D has been used in autoimmune diseases, cancer and osteoporosis (Sintov et al., 2014). Vitamin D deficiency leads to the excessive production of ROS in mitochondria and damages the antioxidant system (Latham et al., 2021). Previous studies have confirmed that vitamin D compounds regulate the proliferation, differentiation, migration and apoptosis of melanocytes and affect T-cell mediated peripheral immune response (Birlea et al., 2008; Kawakami et al., 2014). In H_2_O_2-_induced oxidative stress models in PIG1 and PIG3V cells, vitamin D activated WNT/β-Catenin signaling pathway exerted antioxidant activity, the mechanism included the promotion of GSK3β inactivation, increased β-catenin nuclear translocation and activation of the downstream Nrf2/ARE pathway. In addition, vitamin D passed through up regulation of MITF in a β-catenin pathway dependent manner promoted melanocyte proliferation and protected melanocytes from H_2_O_2-_induced apoptosis by suppressing caspase3 expression (Tang et al., 2018).

## Conclusion and Prospective

In traditional medicine, there are many traditional herbal medicines (Kwon et al., 2018; Xu et al., 2017; Moreira et al., 2012) for the treatment of pigment deficiency. Their extracts significantly improved the activity of tyrosinase (Xu et al., 2017). However, their specific components and mechanism of action are still unclear. Vitiligo is an autoimmune skin disease in which melanocytes are destroyed by autoreactive CD8^+^ T cells, resulting in cutaneous leukoplakia. Depigmented mouse skin lesions with autoimmune features were fitted to a monobenzone-induced vitiligo model (Zhu et al., 2013). Unfortunately, among all natural drugs mentioned in this review, only four natural products (such as Vitexin, baicalin, EGCG and berberine) currently exhibit anti depigmenting pharmacological activity in this model. We mentioned earlier that oxidative stress may be a driver of autoimmune destruction and that intense and persistent oxidative stress leads to apoptosis, damage, and antigen exposure of melanocytes, which in turn trigger autoimmune destruction. Therefore, recently, researchers have paid more attention to the mechanism of action of natural products against oxidative stress in melanocytes. Some natural drugs (such as baicalein, Vitexin, maclurin, etc.) inhibited the damaging effects of H_2_O_2_-induced oxidative stress on melanocytes, even counterstaining depigmented mouse skin lesions. Therefore, it is reasonable to speculate that some of these members contribute to protection against autoimmune destruction. 37 natural products can elevate melanin expression by elevating tyrosinase activity in an *in vitro* melanocyte model *in vivo*, but their contribution to intervening in autoimmune destruction is similarly unknown. The above mentioned vitiligo models are all inducible and cannot fully replicate the disease characteristics of human vitiligo, thus increasing the difficulty of developing new drugs. Genetically edited mice have not been widely promoted (Riding et al., 2019). Several natural drugs have been reported for their use in the clinic. For example: observations from clinical studies have shown that oral Glycyrrhizin Combined with UVB therapy improves active systemic vitiligo, but also exposes minor side effects (Mou et al., 2016). In clinical studies, the overall recolor rate of PUVA (psoralen plus UVA) was only 44% (Bishnoi and Parsad 2018). However, due to the lack of large clinical research, they still have a long way to go before they can be promoted as anti-vitiligo agents (Maleš et al., 2019). A systematic review study showed that vitiligo does not generally produce benefi cial outcomes with the use of topical antioxidants, but the added effect of oral intake cannot be ignored (Speeckaert et al., 2018). Developing new natural products against autoimmune destruction will be a hotspot in the future and also provide a new direction to elucidate the pathogenesis of vitiligo. Vitiligo treatment serves two purposes: 1) Controlling vitiligo disease progression during the active phase and 2) increasing melanocyte production during the stationary phase. We propose the hypothesis that in the future natural drugs with anti-oxidant and anti-autoimmune properties are used in vitiligo progression phase and natural drugs with improved tyrosinase activity are used in stability phase.

In conclusion, strong evidence that natural products can prevent or treat vitiligo is still lacking. Appropriately designed clinical trials are needed to further understand the efficacy of natural products against vitiligo. As an auxiliary means of phototherapy, plant derived compounds with antioxidant properties are becoming an attractive choice for the treatment of vitiligo (Dell'Anna et al., 2007). Natural products (NPs) extracted from plants show the effect of increasing the expression of melanin, and have less side effects on human body, which is of great significance for us to continue to develop natural drugs (Yin et al., 2017).

## Abbreviations

HO-1, heme oxygenase-1; Nrf2, The nuclear factor erythroid 2-like factor 2; NE, Norepinephrine; GCLM, g-glutamylcystine ligase modulatory subunit; GCLC, g-glutamylcystine ligase catalytic subunit; Cyt-4, cytochrome c; ROS, reactive oxygen species; TYR, tyrosinase; TRP-1, tyrosinase-related protein 1; TRP-2, tyrosinase-related protein 2; MITF, microphthalmia-associated transcription factor; MAPK, mitogen-activated protein kinase; ERK, extracellular signal-regulated kinase; JNK, c-Jun N-terminal kinase; cAMP, intracellular cyclic adenosine monophosphate; GSK-3β, glycogen synthase kinase 3β; cRE, cAMP-response element; cREB, cAMP-response element binding protein; PKA, protein kinase A; α-MSH, α melanocyte stimulating hormone; PI3K, phosphoinositide 3-kinase; Akt, protein kinase B; c-Kit, proto-oncogene; TNF-α, tumor necrosis factor alpha; IFN-γ, interferon-γ; ARE, antioxidant response element; SOD, Superoxide dismutase; CAT, catalase; HEMn, normal human epidermal melanocytes; HEKn, normal human epidermal keratinocytes
